# Disulfidptosis: Six Riddles Necessitating Solutions

**DOI:** 10.7150/ijbs.90606

**Published:** 2024-01-20

**Authors:** Xinyu Wang, Chanjuan Zheng, Hui Yao, Yuxuan Guo, Yian Wang, Guangchun He, Shujun Fu, Xiyun Deng

**Affiliations:** 1 Key Laboratory of Translational Cancer Stem Cell Research, Department of Pathophysiology, Hunan Normal University School of Medicine, Changsha, Hunan 410013, China.; 2 Department of Pathology, Xiangtan Central Hospital, Xiangtan, Hunan 411100, China.

**Keywords:** Programmed cell death, Disulfidptosis, Disulfide stress, Actin cytoskeleton, Cellular metabolism

## Abstract

Disulfidptosis occurs as a result of the accumulation of intracellular cystine followed by disulfide stress in actin cytoskeleton proteins due to a reduction of NADPH produced through the pentose phosphate pathway in cells with high expression of SLC7A11. It is a cell death caused by the redox imbalance resulting from the disruption of amino acid metabolism and glucose metabolism. The discovery of disulfidptosis has sparked immense enthusiasm, but there are numerous unresolved issues that need to be addressed. Solutions to these riddles will provide insights into the detailed mechanisms and the pathophysiological relevance of disulfidptosis and utilizing disulfidptosis as an actionable therapeutic target.

The redox homeostasis is crucial for cellular life. When there is insufficient production or excessive consumption of intracellular NADPH, the redox homeostasis within the cell is disrupted 1. This disruption leads to excessive accumulation of intracellular cystine and disulfide stress in actin cytoskeleton proteins, resulting in actin network collapse and eventually induction of disulfidptosis, a novel form of programmed cell death 2. The discovery of disulfidptosis has sparked immense enthusiasm, but there are numerous unresolved issues that need to be addressed in future studies. In this commentary, we discuss six riddles brought up with the discovery of disulfidptosis (**Figure 1**), trying to stimulate further thinking and provide directions for future research.

## Riddle #1: What are the precise mechanisms of disulfidptosis?

The involvement of actin cytoskeleton proteins was identified based on the alterations of the disulfide proteome 2. It is not clear, however, why disulfide stress occurs preferentially in actin cytoskeleton-associated proteins and whether other cytoskeleton-associated proteins such as intermediate filaments, microtubules, or even signaling proteins are also involved in disulfidptosis induction. It is not known why proteins in the endoplasmic reticulum, where significant disulfide bonding occurs due to the oxidized environment 3, are not sensitive to stress-related disulfide bonding. Possibly, cytosolic proteins such as those of the actin cytoskeleton, which typically do not form extensive disulfide bonding due to the reducing environment, might be more redox-sensitive than proteins of other locations in the cell under stress conditions 4.

Indeed, disulfide bonding was also identified in focal adhesion-associated tyrosine kinases of glucose-starved SLC7A11^high^ cells 2. How tyrosine kinase signaling contributes to disulfide stress will be a subject of intense research interest. Moreover, focal adhesion is involved in cancer cell invasion and metastasis 5. The role of the adhesion-invasion-metastasis sequence in disulfidptosis, for example, in the case of high SLC7A11 expression inhibiting metastasis 6, is worthy of further investigation.

## Riddle #2: How is disulfidptosis different from other types of redox-related cell death?

Both disulfidptosis and ferroptosis are related to the balance of two redox regulatory pairs, i.e., cystine *vs.* cysteine and NADP^+^ *vs.* NADPH. It should be noted that cysteine and its metabolic derivative glutathione are important points of connection between disulfidptosis and other redox-related types of cell death, such as cuproptosis and ferroptosis. As a copper chaperone, glutathione protects the cells from copper toxicity and delays cuproptosis 7. Interestingly, these three types of cell death are all redox homeostasis-related. A key factor(s) may exist that serve as a switch for the cell to sway among disulfidptosis, ferroptosis, and cuproptosis. Presumably, this common switch is the cystine *vs.* cysteine pair, which plays a fundamental role in the regulation of redox homeostasis. However, the difference among these cell death types needs to be further determined.

## Riddle #3: What's the relationship between disulfide stress and other forms of post-translational modifications?

Broadly speaking, disulfide bonding is a protein post-translational modification (PTM). What is the relationship between disulfide stress and other forms of PTMs? Interestingly, PTMs such as ubiquitination, glycosylation, and succinylation on GPX4, have been reported, which are involved in ferroptosis regulation 8. Are these similar PTMs also related to disulfide stress and disulfidptosis? And how? Also, are these different PTMs parallel to each other or do they occur sequentially? The answers to these questions will reveal important clues to understanding the relationship between the different PTMs related to cancer.

## Riddle #4: What's the pathophysiological significance of disulfidptosis?

As with many other forms of programmed cell death, a similar question arises whether this novel type of cell death unexceptionally lead to cell death. Or is there a “point-of-no-return” of the disulfidptosis process? More recently, work from the same group led by Prof. Gan demonstrated that oxidative stress exerted differential roles in influencing tumor growth *vs.* metastasis, with high SLC7A11 expression promoting tumor growth but contrarily inhibiting tumor metastasis 6. Perhaps the disulfidptosis machinery is one of the strategies employed by the host to select those cells that are able to adapt to or survive the oxidative environment during cancer metastasis. However, if the cell won't die upon disulfidptosis, it will most likely withstand the stressor and survive better. If that's the case, cancer cell survival after disulfidptosis will cause the severe issue of therapy resistance.

## Riddle #5: What's the predictive value of the disulfidptosis-related genes?

If dysregulation of the disulfidptosis-related pathway is a characteristic change in certain types of cancer, the prognostic value of disulfidptosis can be exploited. There has already been tremendous progress in generating disulfidptosis-related gene signatures to predict prognosis and immunotherapy responses in various types of cancer. Generation of these signatures based on disulfidptosis to predict the prognosis and therapeutic responses will assist in patient selection for proper therapies.

## Riddle #6: What are the therapeutic implications of disulfidptosis?

GLUT1 inhibitor BAY-876 and GLUT1/3 inhibitor KL-11743 to block glucose uptake can effectively induce disulfidptosis in SLC7A11^high^ cancer cells 2. Glucose is widely needed by almost all tissues in the body. Will global GLUT1 inhibition have a widespread impact on normal tissues? To avoid the side effects of glucose depletion, directed delivery of GLUT1-targeted drugs to cancer cells is of utmost importance in this sense.

It has been demonstrated that induction of ferroptosis can remodel the tumor immune microenvironment 9. Does induction of disulfidptosis cause similar alterations in the immune microenvironment? If so, can we combine disulfidptosis inducers such as GLUT1 inhibitors with immunotherapeutic approaches such as PD-1/PD-L1 antibodies to boost more efficient tumor killing? The combination of disulfidptosis-targeting agents and immunotherapies may be an important direction for future research on disulfidptosis.

In summary, the discovery of disulfidptosis has motivated great enthusiasm for disulfidptosis-related research. Since disulfidptosis is a brand-new area, high expectations need to be sustained by demonstrated progress from basic to translational research. The 6 riddles we propose here may not be a complete list of the questions/issues facing disulfidptosis research. Nevertheless, solutions to these riddles will pave the way to the successful translation of the disulfidptosis pathway into a clinically relevant target for the efficient treatment of cancer and other diseases.

## Figures and Tables

**Figure 1:**
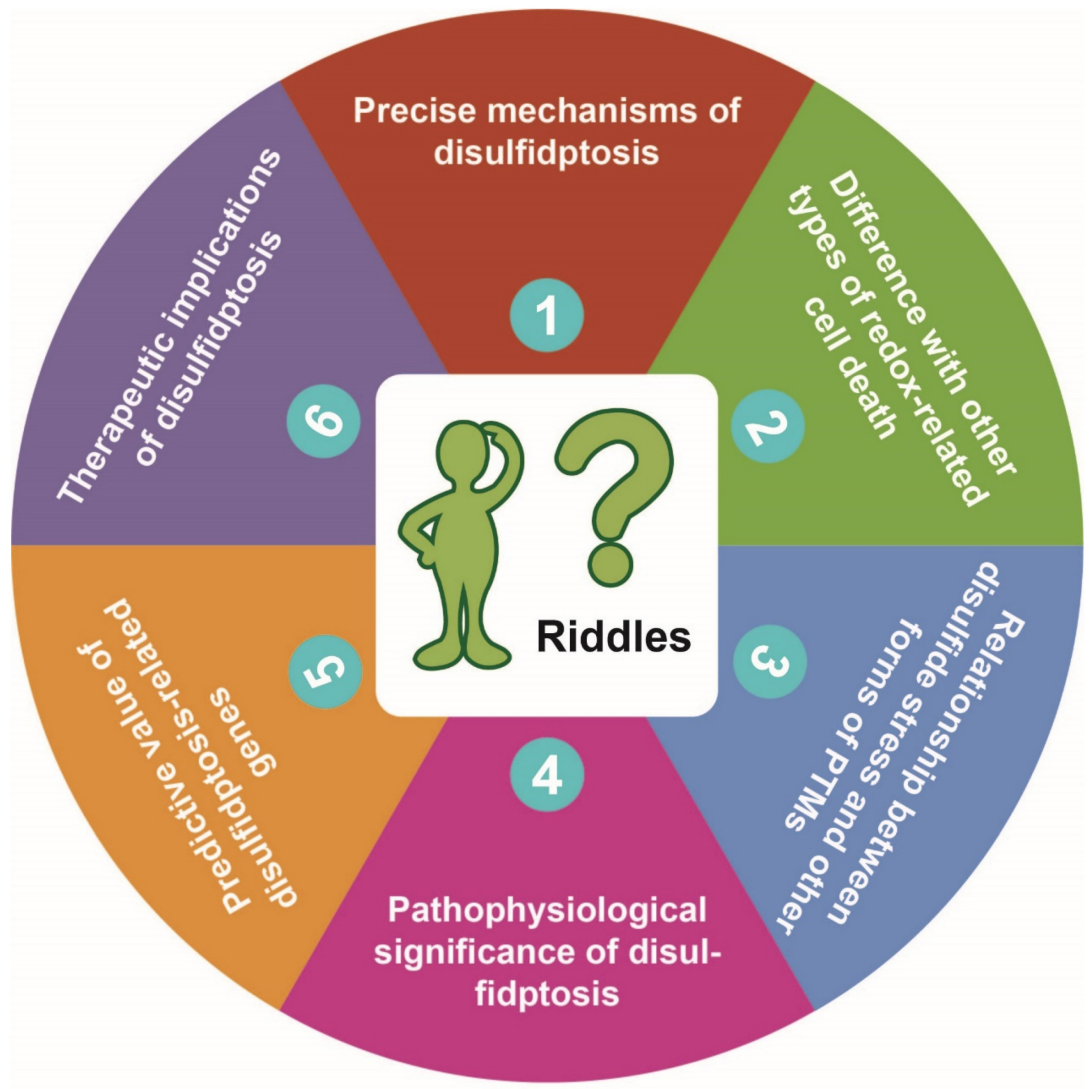
**Proposed six riddles of disulfidptosis.** See text for detailed description of each of the riddles proposed by us.
